# Supplementary material for: A Critical View of the Application of the APEX Software (Aqueous Photochemistry of Environmentally-Occurring Xenobiotics) to Predict Photoreaction Kinetics in Surface Freshwaters
Source: Molecules. 2019 Dec 18;25(1):9. doi: 10.3390/molecules25010009 (PMC7017383; doi:10.3390/molecules25010009)
Supplement: Supplementary file 1 [file molecules-25-00009-s001.zip › 3.2.4_gcc-4.4.0.zip!/3.2.4_gcc-4.4.0/3.2.4_gcc-4.4.0/doc/octave/html/liboctave/Function-Index.html]

Function Index - Octave C++ Classes


Previous: Concept Index,
Up: Top


---

## Function Index

- `absolute_tolerance`: Quadrature
- `absolute_tolerance`: Ordinary Differential Equations
- `add_left`: Collocation Weights
- `add_right`: Collocation Weights
- `AEPBALANCE`: Matrix Factorizations
- `all`: Matrix and Vector Operations
- `alpha`: Collocation Weights
- `any`: Matrix and Vector Operations
- `append`: Matrix and Vector Operations
- `Array2<T>`: Constructors and Assignment
- `Array3<T>`: Constructors and Assignment
- `Array<T>`: Constructors and Assignment
- `balanced_a_matrix`: Matrix Factorizations
- `balanced_b_matrix`: Matrix Factorizations
- `balanced_matrix`: Matrix Factorizations
- `balancing_matrix`: Matrix Factorizations
- `base`: Ranges
- `beta`: Collocation Weights
- `Bounds`: Bounds
- `capacity on Array<T>`: Constructors and Assignment
- `checkelem on Array2<T>`: Constructors and Assignment
- `checkelem on Array3<T>`: Constructors and Assignment
- `checkelem on Array<T>`: Constructors and Assignment
- `checkelem on DiagArray<T>`: Constructors and Assignment
- `CHOL`: Matrix Factorizations
- `chol_matrix`: Matrix Factorizations
- `clear_stop_time`: Ordinary Differential Equations
- `coefficient`: Matrix Factorizations
- `CollocWt`: Collocation Weights
- `cols on Array2<T>`: Constructors and Assignment
- `cols on DiagArray<T>`: Constructors and Assignment
- `column`: Matrix and Vector Operations
- `column_max`: Matrix and Vector Operations
- `column_max_loc`: Matrix and Vector Operations
- `column_min`: Matrix and Vector Operations
- `column_min_loc`: Matrix and Vector Operations
- `columns on Array2<T>`: Constructors and Assignment
- `columns on DiagArray<T>`: Constructors and Assignment
- `ColumnVector`: Matrix and Vector Operations
- `ComplexAEPBALANCE`: Matrix Factorizations
- `ComplexCHOL`: Matrix Factorizations
- `ComplexColumnVector`: Matrix and Vector Operations
- `ComplexDET`: Matrix Factorizations
- `ComplexDiagMatrix`: Matrix and Vector Operations
- `ComplexHESS`: Matrix Factorizations
- `ComplexLU`: Matrix Factorizations
- `ComplexMatrix`: Matrix and Vector Operations
- `ComplexQR`: Matrix Factorizations
- `ComplexRowVector`: Matrix and Vector Operations
- `ComplexSCHUR`: Matrix Factorizations
- `ComplexSVD`: Matrix Factorizations
- `conj`: Matrix and Vector Operations
- `constraint_matrix`: Linear Constraints
- `copy`: Nonlinear Equations
- `copy`: Ordinary Differential Equations
- `copy`: Quadrature
- `cumprod`: Matrix and Vector Operations
- `cumsum`: Matrix and Vector Operations
- `DAE`: Differential Algebraic Equations
- `data on Array<T>`: Constructors and Assignment
- `DefQuad`: Quadrature
- `delete_left`: Collocation Weights
- `delete_right`: Collocation Weights
- `deriv`: Differential Algebraic Equations
- `DET`: Matrix Factorizations
- `determinant`: Matrix and Vector Operations
- `diag`: Matrix and Vector Operations
- `DiagArray<T>`: Constructors and Assignment
- `DiagMatrix`: Matrix and Vector Operations
- `dim1 on Array2<T>`: Constructors and Assignment
- `dim1 on Array3<T>`: Constructors and Assignment
- `dim1 on DiagArray<T>`: Constructors and Assignment
- `dim2 on Array2<T>`: Constructors and Assignment
- `dim2 on Array3<T>`: Constructors and Assignment
- `dim2 on DiagArray<T>`: Constructors and Assignment
- `dim3 on Array3<T>`: Constructors and Assignment
- `EIG`: Matrix Factorizations
- `eigenvalues`: Matrix Factorizations
- `eigenvectors`: Matrix Factorizations
- `elem on Array2<T>`: Constructors and Assignment
- `elem on Array3<T>`: Constructors and Assignment
- `elem on Array<T>`: Constructors and Assignment
- `elem on DiagArray<T>`: Constructors and Assignment
- `eq_constraint_matrix`: Linear Constraints
- `eq_constraint_vector`: Linear Constraints
- `exponent`: Matrix Factorizations
- `extract`: Matrix and Vector Operations
- `fill`: Matrix and Vector Operations
- `first`: Collocation Weights
- `force_restart`: Ordinary Differential Equations
- `fourier`: Matrix and Vector Operations
- `function`: Nonlinear Functions
- `GEPBALANCE`: Matrix Factorizations
- `gradient_function`: Objective Functions
- `HESS`: Matrix Factorizations
- `hess_matrix`: Matrix Factorizations
- `ifourier`: Matrix and Vector Operations
- `imag`: Matrix and Vector Operations
- `inc`: Ranges
- `IndefQuad`: Quadrature
- `ineq_constraint_matrix`: Linear Constraints
- `ineq_constraint_vector`: Linear Constraints
- `init`: Quadrature
- `init`: Nonlinear Equations
- `init`: Ordinary Differential Equations
- `initial_step_size`: Ordinary Differential Equations
- `initialize`: Ordinary Differential Equations
- `initialize`: Differential Algebraic Equations
- `insert`: Matrix and Vector Operations
- `integrate`: Differential Algebraic Equations
- `integrate`: Ordinary Differential Equations
- `integrate`: Differential Algebraic Equations
- `integrate`: Quadrature
- `integrate`: Ordinary Differential Equations
- `integrate`: Quadrature
- `inverse`: Matrix and Vector Operations
- `jacobian_function`: Nonlinear Functions
- `L`: Matrix Factorizations
- `left`: Collocation Weights
- `left_balancing_matrix`: Matrix Factorizations
- `left_included`: Collocation Weights
- `left_singular_matrix`: Matrix Factorizations
- `length on Array<T>`: Constructors and Assignment
- `limit`: Ranges
- `LinConst`: Linear Constraints
- `lower_bound`: Bounds
- `lower_bounds`: Bounds
- `lssolve`: Matrix and Vector Operations
- `LU`: Matrix Factorizations
- `map`: Matrix and Vector Operations
- `Matrix`: Matrix and Vector Operations
- `max`: Matrix and Vector Operations
- `max`: Ranges
- `maximum_step_size`: Ordinary Differential Equations
- `min`: Matrix and Vector Operations
- `min`: Ranges
- `min`: Matrix and Vector Operations
- `minimize`: Nonlinear Programming
- `minimize`: Quadratic Programming
- `minimize`: Nonlinear Programming
- `minimize`: Quadratic Programming
- `minimize`: Nonlinear Programming
- `minimize`: Quadratic Programming
- `minimize`: Nonlinear Programming
- `minimize`: Quadratic Programming
- `minimum_step_size`: Ordinary Differential Equations
- `ncol`: Collocation Weights
- `nelem`: Ranges
- `NLConst`: Nonlinear Constraints
- `NLEqn`: Nonlinear Equations
- `NLEqn_options`: Nonlinear Equations
- `NLFunc`: Nonlinear Functions
- `NLP`: Nonlinear Programming
- `Objective`: Objective Functions
- `objective_function`: Objective Functions
- `ODE`: Ordinary Differential Equations
- `ODE_options`: Ordinary Differential Equations
- `operator !`: Matrix and Vector Operations
- `operator !=`: Matrix and Vector Operations
- `operator () on Array2<T>`: Constructors and Assignment
- `operator () on Array3<T>`: Constructors and Assignment
- `operator () on Array<T>`: Constructors and Assignment
- `operator () on DiagArray<T>`: Constructors and Assignment
- `operator *`: Matrix and Vector Operations
- `operator *`: Constructors and Assignment
- `operator *`: Matrix and Vector Operations
- `operator *`: Constructors and Assignment
- `operator *`: Matrix and Vector Operations
- `operator *`: Constructors and Assignment
- `operator *`: Matrix and Vector Operations
- `operator *`: Constructors and Assignment
- `operator *`: Matrix and Vector Operations
- `operator *`: Constructors and Assignment
- `operator *`: Matrix and Vector Operations
- `operator *`: Constructors and Assignment
- `operator *`: Matrix and Vector Operations
- `operator +`: Matrix and Vector Operations
- `operator +`: Constructors and Assignment
- `operator +`: Matrix and Vector Operations
- `operator +`: Constructors and Assignment
- `operator +`: Matrix and Vector Operations
- `operator +`: Constructors and Assignment
- `operator +`: Matrix and Vector Operations
- `operator +`: Constructors and Assignment
- `operator +`: Matrix and Vector Operations
- `operator +`: Constructors and Assignment
- `operator +`: Matrix and Vector Operations
- `operator +`: Constructors and Assignment
- `operator +`: Matrix and Vector Operations
- `operator +`: Constructors and Assignment
- `operator +`: Matrix and Vector Operations
- `operator +=`: Matrix and Vector Operations
- `operator -`: Matrix and Vector Operations
- `operator -`: Constructors and Assignment
- `operator -`: Matrix and Vector Operations
- `operator -`: Constructors and Assignment
- `operator -`: Matrix and Vector Operations
- `operator -`: Constructors and Assignment
- `operator -`: Matrix and Vector Operations
- `operator -`: Constructors and Assignment
- `operator -`: Matrix and Vector Operations
- `operator -`: Constructors and Assignment
- `operator -`: Matrix and Vector Operations
- `operator -`: Constructors and Assignment
- `operator -`: Matrix and Vector Operations
- `operator -`: Constructors and Assignment
- `operator -`: Matrix and Vector Operations
- `operator -`: Constructors and Assignment
- `operator -`: Matrix and Vector Operations
- `operator -=`: Matrix and Vector Operations
- `operator /`: Matrix and Vector Operations
- `operator /`: Constructors and Assignment
- `operator /`: Matrix and Vector Operations
- `operator /`: Constructors and Assignment
- `operator /`: Matrix and Vector Operations
- `operator /`: Constructors and Assignment
- `operator /`: Matrix and Vector Operations
- `operator /`: Constructors and Assignment
- `operator /`: Matrix and Vector Operations
- `operator <<`: Ranges
- `operator <<`: Matrix and Vector Operations
- `operator <<`: Collocation Weights
- `operator <<`: Matrix Factorizations
- `operator <<`: Matrix and Vector Operations
- `operator <<`: Bounds
- `operator <<`: Matrix Factorizations
- `operator <<`: Matrix and Vector Operations
- `operator <<`: Matrix Factorizations
- `operator <<`: Matrix and Vector Operations
- `operator <<`: Matrix Factorizations
- `operator <<`: Matrix and Vector Operations
- `operator <<`: Matrix Factorizations
- `operator <<`: Matrix and Vector Operations
- `operator <<`: Linear Constraints
- `operator <<`: Matrix Factorizations
- `operator <<`: Matrix and Vector Operations
- `operator <<`: Matrix Factorizations
- `operator =`: Matrix Factorizations
- `operator =`: Matrix and Vector Operations
- `operator =`: Collocation Weights
- `operator =`: Linear Constraints
- `operator =`: Matrix Factorizations
- `operator =`: Matrix and Vector Operations
- `operator =`: Matrix Factorizations
- `operator =`: Objective Functions
- `operator =`: Matrix Factorizations
- `operator =`: Nonlinear Equations
- `operator =`: Nonlinear Programming
- `operator =`: Nonlinear Constraints
- `operator =`: Matrix Factorizations
- `operator =`: Matrix and Vector Operations
- `operator =`: Matrix Factorizations
- `operator =`: Quadrature
- `operator =`: Nonlinear Functions
- `operator =`: Matrix and Vector Operations
- `operator =`: Matrix Factorizations
- `operator =`: Ordinary Differential Equations
- `operator =`: Matrix and Vector Operations
- `operator =`: Nonlinear Equations
- `operator =`: Bounds
- `operator =`: Matrix Factorizations
- `operator =`: Matrix and Vector Operations
- `operator = on Array2<T>`: Constructors and Assignment
- `operator = on Array3<T>`: Constructors and Assignment
- `operator = on Array<T>`: Constructors and Assignment
- `operator = on DiagArray<T>&`: Constructors and Assignment
- `operator ==`: Matrix and Vector Operations
- `operator >>`: Ranges
- `operator >>`: Matrix and Vector Operations
- `P`: Matrix Factorizations
- `print_range`: Ranges
- `prod`: Matrix and Vector Operations
- `product`: Matrix and Vector Operations
- `product`: Constructors and Assignment
- `product`: Matrix and Vector Operations
- `product`: Constructors and Assignment
- `product`: Matrix and Vector Operations
- `product`: Constructors and Assignment
- `product`: Matrix and Vector Operations
- `Q`: Matrix Factorizations
- `QP`: Quadratic Programming
- `QR`: Matrix Factorizations
- `quad`: Collocation Weights
- `Quad`: Quadrature
- `Quad_options`: Quadrature
- `quad_weights`: Collocation Weights
- `quotient`: Matrix and Vector Operations
- `quotient`: Constructors and Assignment
- `quotient`: Matrix and Vector Operations
- `quotient`: Constructors and Assignment
- `quotient`: Matrix and Vector Operations
- `R`: Matrix Factorizations
- `Range`: Ranges
- `real`: Matrix and Vector Operations
- `relative_tolerance`: Quadrature
- `relative_tolerance`: Ordinary Differential Equations
- `resize`: Bounds
- `resize`: Nonlinear Equations
- `resize`: Collocation Weights
- `resize`: Linear Constraints
- `resize on Array2<T>`: Constructors and Assignment
- `resize on Array3<T>`: Constructors and Assignment
- `resize on Array<T>`: Constructors and Assignment
- `resize on DiagArray<T>`: Constructors and Assignment
- `right`: Collocation Weights
- `right_balancing_matrix`: Matrix Factorizations
- `right_included`: Collocation Weights
- `right_singular_matrix`: Matrix Factorizations
- `roots`: Collocation Weights
- `row`: Matrix and Vector Operations
- `row_max`: Matrix and Vector Operations
- `row_max_loc`: Matrix and Vector Operations
- `row_min`: Matrix and Vector Operations
- `row_min_loc`: Matrix and Vector Operations
- `rows on Array2<T>`: Constructors and Assignment
- `rows on DiagArray<T>`: Constructors and Assignment
- `RowVector`: Matrix and Vector Operations
- `SCHUR`: Matrix Factorizations
- `schur_matrix`: Matrix Factorizations
- `second`: Collocation Weights
- `set_absolute_tolerance`: Ordinary Differential Equations
- `set_absolute_tolerance`: Quadrature
- `set_alpha`: Collocation Weights
- `set_base`: Ranges
- `set_beta`: Collocation Weights
- `set_bound`: Bounds
- `set_bounds`: Bounds
- `set_constraint_matrix`: Linear Constraints
- `set_default_options`: Nonlinear Equations
- `set_default_options`: Ordinary Differential Equations
- `set_default_options`: Quadrature
- `set_function`: Nonlinear Functions
- `set_gradient_function`: Objective Functions
- `set_inc`: Ranges
- `set_initial_step_size`: Ordinary Differential Equations
- `set_jacobian_function`: Nonlinear Functions
- `set_left`: Collocation Weights
- `set_limit`: Ranges
- `set_lower_bound`: Bounds
- `set_lower_bounds`: Bounds
- `set_maximum_step_size`: Ordinary Differential Equations
- `set_minimum_step_size`: Ordinary Differential Equations
- `set_objective_function`: Objective Functions
- `set_relative_tolerance`: Quadrature
- `set_relative_tolerance`: Ordinary Differential Equations
- `set_right`: Collocation Weights
- `set_states`: Nonlinear Equations
- `set_stop_time`: Ordinary Differential Equations
- `set_tolerance`: Nonlinear Equations
- `set_upper_bound`: Bounds
- `set_upper_bounds`: Bounds
- `singular_values`: Matrix Factorizations
- `size`: Ordinary Differential Equations
- `size`: Nonlinear Programming
- `size`: Nonlinear Equations
- `size`: Bounds
- `solve`: Matrix and Vector Operations
- `solve`: Nonlinear Equations
- `solve`: Matrix and Vector Operations
- `solve`: Nonlinear Equations
- `solve`: Matrix and Vector Operations
- `sort`: Ranges
- `stack`: Matrix and Vector Operations
- `state`: Ordinary Differential Equations
- `states`: Nonlinear Equations
- `sum`: Matrix and Vector Operations
- `sumsq`: Matrix and Vector Operations
- `SVD`: Matrix Factorizations
- `time`: Ordinary Differential Equations
- `tolerance`: Nonlinear Equations
- `transpose`: Matrix and Vector Operations
- `U`: Matrix Factorizations
- `unitary_hess_matrix`: Matrix Factorizations
- `unitary_matrix`: Matrix Factorizations
- `upper_bound`: Bounds
- `upper_bounds`: Bounds
- `value`: Matrix Factorizations
- `value_will_overflow`: Matrix Factorizations
- `value_will_underflow`: Matrix Factorizations
- `width`: Collocation Weights
- `xelem on Array<T>`: Constructors and Assignment
